# Using single-sample networks and genetic algorithms to identify radiation-responsive genes in rice affected by heavy ions of the galactic cosmic radiation with different LET values

**DOI:** 10.3389/fpls.2024.1457587

**Published:** 2024-11-08

**Authors:** Yan Zhang, Wei Wang, Meng Zhang, Binquan Zhang, Shuai Gao, Meng Hao, Dazhuang Zhou, Lei Zhao, Guenther Reitz, Yeqing Sun

**Affiliations:** ^1^ Institute of Environmental Systems Biology, College of Environmental Science and Engineering, Dalian Maritime University, Dalian, Liaoning, China; ^2^ National Space Science Center, Chinese Academy of Sciences, Beijing, China; ^3^ Consultant German Aerospace Center, Aerospace Medicine, Radiobiology Department, Köln, Germany; ^4^ School of Radiation Medicine and Protection, Soochow University, Suzhou, China

**Keywords:** spaceflight, space radiation, heavy ions, linear energy transfer, LET regression model, gene interaction pattern

## Abstract

**Introduction:**

Heavy ions of the galactic cosmic radiation dominate the radiation risks and biological effects for plants under spaceflight conditions. However, the biological effects and sensitive genes caused by heavy ions with different linear energy transfer (LET) values have not been thoroughly studied.

**Methods:**

To comprehensively analyze the biological effects of heavy ions with different LET values on rice under spaceflight conditions, we utilized the Shijian-10 recoverable satellite (SJ-10) to transport the dehydrated rice seeds on a 12.5-day mission in a 252 km low Earth orbit (LEO), and obtained rice plants hit by individual heavy ions with LET values ranging from 18 keV/μm to 213 keV/μm. The transcriptome and methylation sequencing were conducted on above plants, and a bioinformatics pipeline based on single-sample networks (SSNs) and genetic algorithms (GA) was developed to analyze the multi-omics expression profiles in this work. Note that SSNs can depict the gene interaction patterns within a single sample. The LET regression models were constructed from both gene expression and interaction pattern perspectives respectively, and the radiation response genes that played significant roles in the models were identified. We designed a gene selection algorithm based on GA to enhance the performance of LET regression models.

**Results:**

The experimental results demonstrate that all our models exhibit excellent regression performance (R2 values close to 1), which indicates that both gene expressions and interaction patterns can reflect the molecular changes caused by heavy ions with different LET values. LET-related genes (genes exhibiting strong correlation with LET values) and radiation-responsive genes were identified, primarily involved in DNA damage and repair, oxidative stress, photosynthesis, nucleic acid metabolism, energy metabolism, amino acid/protein metabolism, and lipid metabolism, etc. DNA methylation plays a crucial role in responding to heavy ions stressors and regulates the aforementioned processes.

**Discussion:**

To the best of our knowledge, this is the first study to report the multi-omics changes in plants after exposure to heavy ions with different LET values under spaceflight conditions.

## 1 Introduction

Space radiation primarily consists of high-energy protons from solar particle events (SPE), heavy ions from galactic cosmic rays (GCR), and secondary particles produced through interactions with spacecraft shielding (Chancellor et al., 2014). Its ionization of molecules, cells, and tissues, along with the consequent initial biological effects, significantly deviate from those induced by typical terrestrial radiation (Durante and Cucinotta, 2008). Compared to other types of radiation, heavy ions are high-linear energy transfer (LET) particles that dominate the radiation risk and biological effects for astronauts and biological samples (Benton and Benton, 2001; Townsend and Fry, 2002; Thomas, 2004; Lushbaugh, 1990). Heavy ions create column-like tracks with intense ionization as they pass through a medium, causing damage to numerous cells (Zhou et al., 2018). As a unique abiotic stress factor, heavy ions can exert significant effects on plants. Our previous research indicated that mutations induced by both spaceflight and heavy ion radiation were widespread across the rice genome, encompassing coding region and repeated region (Shi et al., 2014). Moreover, after treating rice with high-energy heavy ion beams, a significant number of SNPs and InDels, as well as genetic variations related to grain type and heading date, were discovered (Wen et al., 2023). However, there are few reports on the multi-omics (such as transcriptomics and methylation) changes in plants after exposure to heavy ions with different LET values under spaceflight conditions.

In recent years, it has been confirmed that spaceflight induces stress responses in rice seeds, leading to changes in protein levels, transcription levels, and methylation levels during the planting process after returning to the ground (Zeng et al., 2021). There have been some studies on omics-level changes in plants under spaceflight conditions. At the transcriptome level, Cui et al. previously discovered that spaceflight affects the expression level of key enzyme genes in the photosynthetic pathway in rice (Cui et al., 2019). Manian et al. used Pearson correlation-based gene regulatory network analysis on the transcriptome profile of *Arabidopsis thaliana* under spaceflight conditions, explaining the DNA damage response processes induced by space stressors (Manian et al., 2021). At the methylation level, the previous work of our team confirmed that spaceflight caused the methylation of the rice genome, and the changes in methylation were more obvious in the representative variants (Sun et al., 2019). Of note, gene expression related to physiological adaptation to spaceflight is partially regulated by methylation strategies (Zhou et al., 2019). Research indicates that DNA methylation triggers the expression of various abiotic stress genes in *Arabidopsis thaliana* progeny, including stress response, cold response, cell wall remodeling, nitrate metabolism, plant hormones, and Ca2+ signal transduction (Xu et al., 2021). Although omics analyses have yielded many valuable insights into plant stress responses under spaceflight conditions, the biological effects in plants hit by heavy ions still require further examination.

More recently, there has been a growing recognition that phenotypic changes in organisms, often driven by complex gene networks, cannot be fully attributed to individual genes in isolation (Kuijjer et al., 2019). Typically, gene networks are constructed from multiple samples, overlooking individual-specific information (Huang et al., 2021). To address this issue, the concept of single-sample network (SSN) has been proposed. The SSN method involves constructing a separate network for each sample, where nodes represent genes and edges represent relationships between genes (Zhang et al., 2024a; Zhang et al., 2024b). When combined with the protein-protein interaction (PPI) network, SSNs can depict the gene interaction patterns within a single sample (Zhang et al., 2024a; Zhang et al., 2024b). In a recent study (Zhang et al., 2024a), we built individual SSNs for 301 spaceflight mouse samples from the GeneLab platform, and identified 20 sensitive genes for space radiation based on dose-response models constructed from gene interaction patterns. Besides, using the SSN method, we also found that mice exposed to radiation doses across three intervals (4.66~7.14 mGy, 7.592~8.295 mGy, 8.49~22.099 mGy) showed comparable patterns of gene interactions (Zhang et al., 2024b). The above results indicate that SSNs can reflect molecular changes caused by space radiation with different physical parameters. Therefore, in this work, we constructed LET regression models from gene expression levels and interaction patterns respectively, and further identified the genes responsive to the LET values of GCR HZEs.

Identifying radiation-responsive genes is an important approach to understand the biological effects of ionizing radiation on plants. However, due to the difficulty in obtaining biological samples hit by individual GCR HZEs, the biological effects and sensitive genes caused by GCR HZEs with different LET values have not been thoroughly studied. In the present study, we utilized the Shijian-10 recoverable satellite (SJ-10) to transport the dehydrated rice seeds on a 12.5-day mission in a 252 km low Earth orbit (LEO). In the SJ-10 experiment, we used a biostack to conduct accurate LET measurement for GCR HZEs that hit biosamples, and successfully obtained rice plants hit by individual GCR HZEs. Note that the detailed design of the biostack and the measurement of LET values have been thoroughly described in our previous publications (Zhou et al., 2018). We performed transcriptome and methylation sequencing on samples hit by individual GCR HZEs with different LET values, and developed a bioinformatics pipeline based on SSNs and genetic algorithms (GA) to analyze the multi-omics expression profiles. The LET regression models were constructed and the radiation response genes that play significant roles in the models were identified. Overall, the primary objective of the present study was to comprehensively analyze the biological effects of heavy ions with different LET values on rice under spaceflight conditions, specifically including: 1) constructing LET regression models from both gene expression and interaction pattern perspectives; 2) identifying LET-related genes and radiation-responsive genes, and analyzing the biological processes they are involved in; and 3) exploring the role of DNA methylation in plant responses to heavy ion stressors.

## 2 Materials and methods

### 2.1 Spaceflight carrying and measurement of LET values

As mentioned above, SJ-10 was utilized to transport the dehydrated seeds of *Oryza. sativa L.* spp. *Japonica, var Nipponbare* on a 12.5-day mission in a 252 km LEO. The ground control group was processed synchronously with the spaceflight group. We used biostack (Zhou et al., 2018; Duan and Long, 2019) to identify the embryos hit by single high LET heavy ions. Biological layers of rice seeds were sandwiched between nuclear track detectors (CR-39), which were positioned in fixed spatial relation to the seeds. After the satellite returned to Earth, we chemically etched the CR-39, scanned the nuclear tracks, and finally determined the LET of each particle. To note, only seeds with embryos hit by a single heavy ion larger than 10 keV/μm were studied in this work. For details on the measurement of LET values, please refer to our previous studies (Zhou et al., 2018).

### 2.2 Rice planting and sequencing analysis

**Figure 1:**
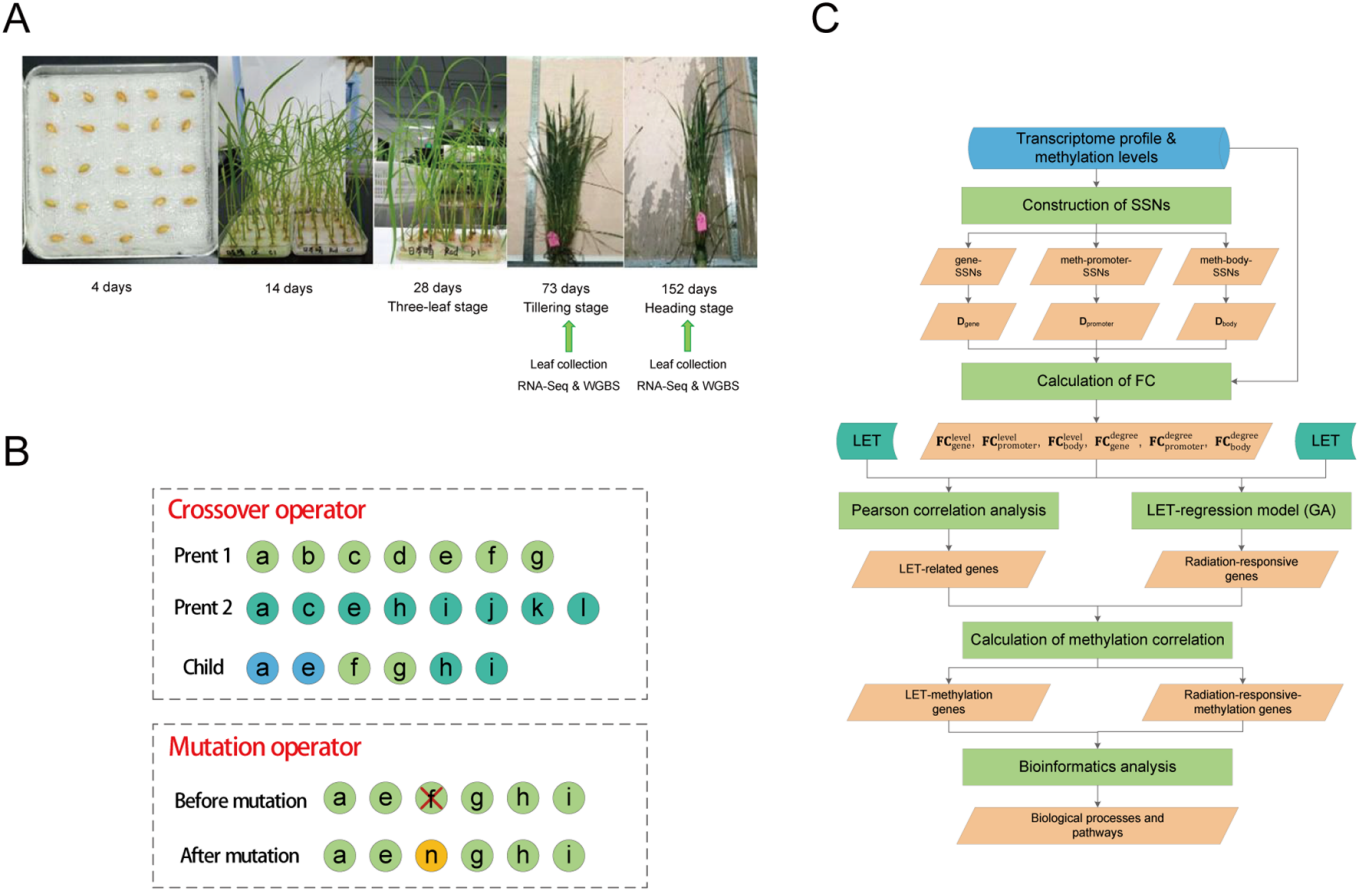
Sample treatment and model construction. **(A)** Rice planting and sequencing analysis. **(B)** Crossover and mutation operations in genetic algorithm. A circle represents a gene. In crossover operation, light green circles represent genes from parent 1, dark green circles represent genes from parent 2, and blue circles represent genes shared by both parents. In mutation operation, the yellow circle represents newly added genes. **(C)** Schematic representation of the analysis workflow in this study. SSNs were constructed separately using the processed transcriptome profile and methylation levels, and three degree-matrices were obtained. Then, the FC of each gene was calculated from the perspectives of both gene expression levels (methylation levels) and degrees, respectively. LET-related genes and radiation-responsive genes were identified through the construction of the LET-regression models and Pearson correlation analysis, and the correlation between these genes and methylation was calculated. Finally, GO/KEGG enrichment analysis and ClueGO analysis were used to explore the biological processes and pathways associated with these genes. Radiation-responsive-methylation genes refer to genes whose expression levels (degrees) are significantly correlated with their corresponding methylation levels (degrees).

A total of 103 seeds were hit by single GCR HZEs (the LET values were shown in **Supplementary Table S1**). On December 15, 2017 (606 days after the satellite returned to Earth), the seeds in ground control and spaceflight group were soaked in 2ml of deionized water at 25°C in darkness for 4 days (**Figure 1A**). On December 19, 2017, the seeds were transferred into petri dishes and placed in a climate-controlled chamber under conditions of 28°C/25°C (day/night), a 14h/10h (day/night) photoperiod, and a light intensity of 300 μmol·m^-2^·s^-1^ for cultivation. After 30 days, the grown plants were transferred to suitable tanks for further growth. Throughout the cultivation process (**Figure 1A**), Yoshida culture solution was regularly replaced until the rice reached maturity.

We randomly selected three ground control plants, 21 plants hit by single GCR HZEs, and one plant that was not hit by any heavy ions during spaceflight for RNA sequencing (RNA-Seq) (**Figure 1A**) (the LET values are shown in **Table 1**). Note that the LET values for the plants in the spaceflight group range between 0 keV/μm and 250.8219 keV/μm. To observe the biological changes in rice at different developmental stages, leaves were collected at the tillering stage (73 days after soaking seeds) and heading stage (152 days after soaking seeds) (**Figure 1A**), respectively. Of note, the leaf samples from the tillering stage and heading stage were taken from the same plant. Moreover, to observe the methylation changes in rice under different LET conditions, we conducted whole-genome bisulfite sequencing (WGBS) on leaves in the tillering and heading stages of eight spaceflight plants and three ground control plants, respectively (**Table 1** and **Figure 1A**). The detailed steps of RNA-Seq and WGBS are as follows:

**Table 1:** LET values of sequenced plants. GC stands for ground control group, and SF stands for spaceflight group.

ID	LET (keV/μm)	RNA-Seq	WGBS
GC1		√	√
GC2		√	√
GC3		√	√
SF1	0	√	√
SF2	18.2679	√	
SF3	19.8511	√	
SF4	20.4151	√	
SF5	52.7435	√	√
SF6	53.7153	√	√
SF7	54.9614	√	
SF8	70.9111	√	
SF9	74.8003	√	
SF10	77.7099	√	
SF11	100.74	√	√
SF12	107.6414	√	√
SF13	107.6414	√	
SF14	136.8396	√	√
SF15	136.8545	√	
SF16	159.4854	√	
SF17	184.0827	√	√
SF18	186.1181	√	√
SF19	206.4886	√	
SF20	213.3132	√	
SF21	248.0167	√	
SF22	250.8219	√	

#### 2.2.1 The extraction of genomic DNA and total RNA

We extracted Genomic DNA from rice leaves using a universal genomic DNA extraction kit (TaKaRa, Dalian, China). Total RNA was isolated and purified from rice leaves using Trizol reagent (Invitrogen, Carlsbad, USA) according to the manufacturer’s protocol. The purity of the DNA and RNA was assessed using Nanodrop (IMPLEN, München, Germany), and the DNA/RNA concentration was accurately quantified with Qubit (Life Technologies, Carlsbad, USA). Finally, we precisely evaluated RNA integrity using Agilent 2100 (Agilent Technologies, Palo Alto, USA).

#### 2.2.2 WGBS library construction and sequencing

We used the Covaris S220 to randomly shear the genomic DNA into 200-300 bp fragments, followed by bisulfite treatment and PCR amplification to obtain the final DNA library. After pooling the different libraries according to their effective concentrations and the output of target data, Illumina HiSeq/MiSeq sequencing was performed. Finally, we used Bismark (Krueger and Andrews, 2011) (Version 0.16.3) to align the methylation data against the rice reference genome. Note that the reference genome was sourced from The Rice Annotation Project Database (Sakai et al., 2013) (https://rapdb.dna.affrc.go.jp/), with the version being IRGSP-1.0.

#### 2.2.3 mRNA library construction and sequencing

mRNA was enriched using VAHTS mRNA CaptureBeads (N401-01/02) (vazyme, Nanjing, China), which bound to the poly(A) tails of mRNA through A-T base pairing. Next, we added fragmentation buffer to shear the mRNA into shorter fragments. Using the fragmented mRNA as a template, we synthesized single-stranded cDNA using random hexamers as primers. Then, we added buffer, dNTPs, and DNA polymerase I to synthesize double-stranded cDNA, and purified the double-stranded cDNA using AMPure XP beads (Beckman Coulter, USA). Finally, PCR enrichment was performed to obtain the final cDNA library.

After pooling the different libraries according to their effective concentrations and the output of target data, HiSeq sequencing was performed. The raw image files generated by high-throughput sequencing were processed through base calling analysis to convert them into sequencing reads, which were stored in FASTQ format. Then, clean reads were obtained after filtering, which were mapped to the rice reference genome (Version IRGSP-1.0) using HISAT (Kim et al., 2015) (Version 2.0.4). Finally, the fragments per kilobase of transcript per million mapped reads (FPKM) value of each gene was calculated by HTSeq (Putri et al., 2022) (Version v0.6.1) (with the “union” mode for counting). Note that the FPKM values of all genes are shown in **Supplementary Table S2**.

### 2.3 Processing of multi-omics data

By aligning with the gene annotation file (from plants.ensembl.org), we calculated the average methylation levels (*ML*) for both the gene body region and the promoter region (upstream 2000 bp) for each gene (Equation 1).


(1) ML=mC/(mC+umC)

Where mC represents the number of reads supporting methylation in a gene region, and umC represents the number of reads supporting non-methylation in a gene region (the methylation levels of all genes in the gene bodies and promoters are shown in **Supplementary Table S3**). In addition, for the transcriptome profile, genes with zero expression levels were removed.

### 2.4 Construction of single-sample networks in multi-omics levels

Once we acquired the gene expression levels and methylation levels for all samples, we utilized LIONESS to generate three separate SSNs (gene-SSN, meth-promoter-SSN, meth-body-SSN) for each sample. Please refer to our previous works (Zhang et al., 2024a; Zhang et al., 2024b) for detailed methods on constructing the SSNs. Of note, the method for constructing the methylation-SSNs is analogous to that of the transcriptome-SSNs, with the distinction being that the input shifts from gene expression levels to methylation levels. To delineate gene interaction patterns within individuals, we overlapped the gene-SSNs, meth-promoter-SSNs, and meth-body-SSNs generated by LIONESS with the PPI network, preserving only the nodes and edges shared between the SSNs and PPI network. The PPI network was sourced from Search Tool for the Retrieval of Interacting Genes (STRING) (Szklarczyk et al., 2023) (Version 12.0) (string-db.org), retaining only experimentally confirmed gene interactions.

The features of genes (nodes) were extracted from the SSNs using degrees. In general, the degree of a node in undirected graphs (like SSNs) refers to the number of its neighbors. We respectively merged the genes contained in the gene-SSNs, meth-promoter-SSNs, and meth-body-SSNs to form three degree-matrices (D_gene_, D_promoter_, D_body_) for the tillering and heading stages. For a specific gene, it corresponds to a vector d (Equation 2) in the matrix.


(2) $$d=\left(d_{1},\;d_{2},\;\cdots ,\;d_{n}\right)$$

Where, $d_{i}$ denotes the degree in the *i*-th SSN (if this gene is not present in the *i*-th SSN, then $d_{i}=0$ ). *n* represents the number of SSNs. In D_gene_, n=25 ; in D_promoter_ and D_body_, n=11 .

### 2.5 Identification of LET-related genes

The fold change (FC) of each gene was calculated from the perspectives of gene expression levels (methylation levels) and degrees respectively. For the expression levels (methylation levels), the FC was calculated according to Equation 3.


(3) $$FC_{i,s}^{\text{level}}=\frac{g_{i,s}^{\text{SF}}}{\text{mean}\left(g_{i}^{\text{GC}}\right)}$$

$FC_{i,s}$ represents the FC value of gene *i* in sample *s*. $g_{i,s}^{\text{SF}}$ represents the expression level or methylation level of gene *i* in sample *s*. $g_{i}^{\text{GC}}$ represents the expression level or methylation level of gene *i* in the ground control samples corresponding to *s*. For the degrees, since the $\text{mean}\left(g_{i}^{\text{GC}}\right)$ might be 0, we made slight modifications to Equation 3 (Equation 4).


(4) $$FC_{i,s}^{\text{degree}}=\frac{g_{i,s}^{\text{SF}}+1}{\text{mean}\left(g_{i}^{\text{GC}}+1\right)}$$

Thus far, we have obtained six FC-matrices ( $FC_{\text{gene}}^{\text{level}}$ , $FC_{\text{promoter}}^{\text{level}}$ , $FC_{\text{body}}^{\text{level}}$ , $FC_{\text{gene}}^{\text{degree}}$ , $FC_{\text{promoter}}^{\text{degree}}$ , and $FC_{\text{body}}^{\text{degree}}$ ) for the tillering and heading stages respectively. Next, we calculated Pearson correlation coefficient (PCC) between the FC vectors of each gene and LET values. For expression levels (methylation levels), genes with “ |PCC|>0.5 and p<0.05 ” were defined as LET-related genes; for degrees, genes with “ p<0.05 ” were defined as LET-related genes.

### 2.6 Identification of radiation-responsive genes using GA

To accurately identify radiation-responsive genes, we used our recently developed method (Zhang et al., 2024a) to construct some LET-regression models based on GA. In simple terms, we used GA to select the optimal combination of genes, enabling the LET-regression model to achieve the best performance. Clearly, the genes selected by the GA are the radiation-responsive genes.

The regression models were constructed for samples from the tillering stage and heading stage respectively. The input features of the model were $FC_{\text{gene}}^{\text{level}}$ , $FC_{\text{gene}}^{\text{degree}}$ , and the target variable (*Y*) was the LET values (**Table 1**). Note that the model with $FC_{\text{gene}}^{\text{level}}$ as input was referred to as the “expression-model”, and the model with $FC_{\text{gene}}^{\text{degree}}$ as input was referred to as the “degree-model”. The regression model was multiple linear regression (MLR). The fitting process of MLR mainly involves estimating the regression coefficients using the ordinary least squares (OLS) method, which minimizes the sum of squared errors between the model’s predicted values and the actual values (*Y*). The specific steps are as follows:

Assuming there is a target variable *Y* and *p* independent variables $X_{1},\;X_{2},\;\cdots X_{p}$ (FC-matrices in this paper), and the form of the regression model is:


(5) $$\text{Y}=\beta_{0}+\beta_{1}{\text{X}}_{1}+\beta_{2}{\text{X}}_{2}+\cdots \beta_{p}{\text{X}}_{\text{p}}$$

The error between the predicted values and the actual values is:


(6) $$e=\sum_{i=1}^{n}{\left(y_{i}-\hat{y_{i}}\right)}^{2}$$

Where, n represents the number of samples. The goal of OLS is to find a set of *β* that minimizes the sum of squared errors (*e*) above.

The training strategy was leave-one-out cross-validation (LOOCV). In LOOCV, one sample was used as the validation set, and the remaining samples were used as the training set. This process was repeated for each sample in the dataset, ensuring that each sample was used once as the validation set. The model’s performance was then averaged over all iterations. The fitness function of the GA was the *R* ^2^ calculated from LOOCV.

To identify the most LET-responsive genes and reduce redundant genes, we improved our previous algorithm (Zhang et al., 2024a) to control the number of genes included in the models. The specific modifications are as follows:

#### 2.6.1 Initialization

In the initialization, the number of features (genes) selected by each solution (individual) was randomly generated between 1 and 50.

#### 2.6.2 Crossover operator

We combined the features included by both parents and then randomly selected some features (**Figure 1B**). If the total number of features from both parents exceeded 50, the number of features in the offspring was randomly generated within the range [1, 50]. If the total number of features from both parents was less than 50, the number of features in the offspring was randomly generated within the range [1, *m*], where *m* was the total number of features from both parents. It should be noted that this crossover strategy ensured that the selected genes’ count remained between 1 and 50.

#### 2.6.3 Mutation operator

To maintain a consistent number of features (genes) in each individual, during the mutation operation, we first randomly removed one feature (gene) from the current individual. Then, another feature from the remaining unselected features was randomly selected to incorporate into the current individual (**Figure 1B**).

To note, all other operations and parameter settings in this work remained consistent with our previous research (Zhang et al., 2024a). The code for our GA model was stored in the GitHub (https://github.com/Zhangyan-DMU/Radiation-Responsive-Gene/tree/main).

### 2.7 Bioinformatics analysis of radiation-responsive genes

In order to investigate the biological significance of radiation-responsive genes (LET-related genes) and further elucidate the biological effects of heavy ions on rice, we conducted a series of bioinformatics analyses on these genes.

Gene Ontology (GO) enrichment analysis was performed on the radiation-responsive genes (LET-related genes) using online tools provided by GENE ONTOLOGY (Thomas et al., 2022) (Release 2024-06-17) (geneontology.org). The species was set to “*Oryza sativa*”, the Annotation Data Set was set to “GO biological process complete”, and the Test Type was set to “Fisher’s Exact”. Significant biological processes (P-value<0.05) were selected for further investigation.

Kyoto Encyclopedia of Genes and Genomes (KEGG) enrichment analysis was performed on the The Database for Annotation, Visualization and Integrated Discovery (DAVID) (Sherman et al., 2022) (Version v2024q1) (david.ncifcrf.gov). The Identifier was set to “ENSEMBL_GENE_ID”, the List Type was set to “Gene List”, the species was set to “Oryza sativa Japonica”, and the “Functional Annotation Tool” was used for analysis. ClueGO (Bindea et al., 2009) (Version 2.5.10) in Cytoscape was used to query pathways associated with radiation-responsive genes (LET-related genes). The species was set to “*Oryza sativa*”, the database was set to “KEGG”, and all the queried pathways were included in the results.

Additionally, we utilized STRING (Szklarczyk et al., 2023) (Version 12.0) to construct the PPI networks for LET-related genes and identified hub genes with high degrees. We selected “Multiple proteins” for the search, chose “*Oryza sativa Japonica*” as the species, and left all other parameters at their default values. Of note, all inputs for the above tools were Rice Annotation Project (RAP) IDs.

The overall analysis workflow of this study is shown in **Figure 1C**.

## 3 Results

### 3.1 LET-related genes and their biological functions

**Figure 2:**
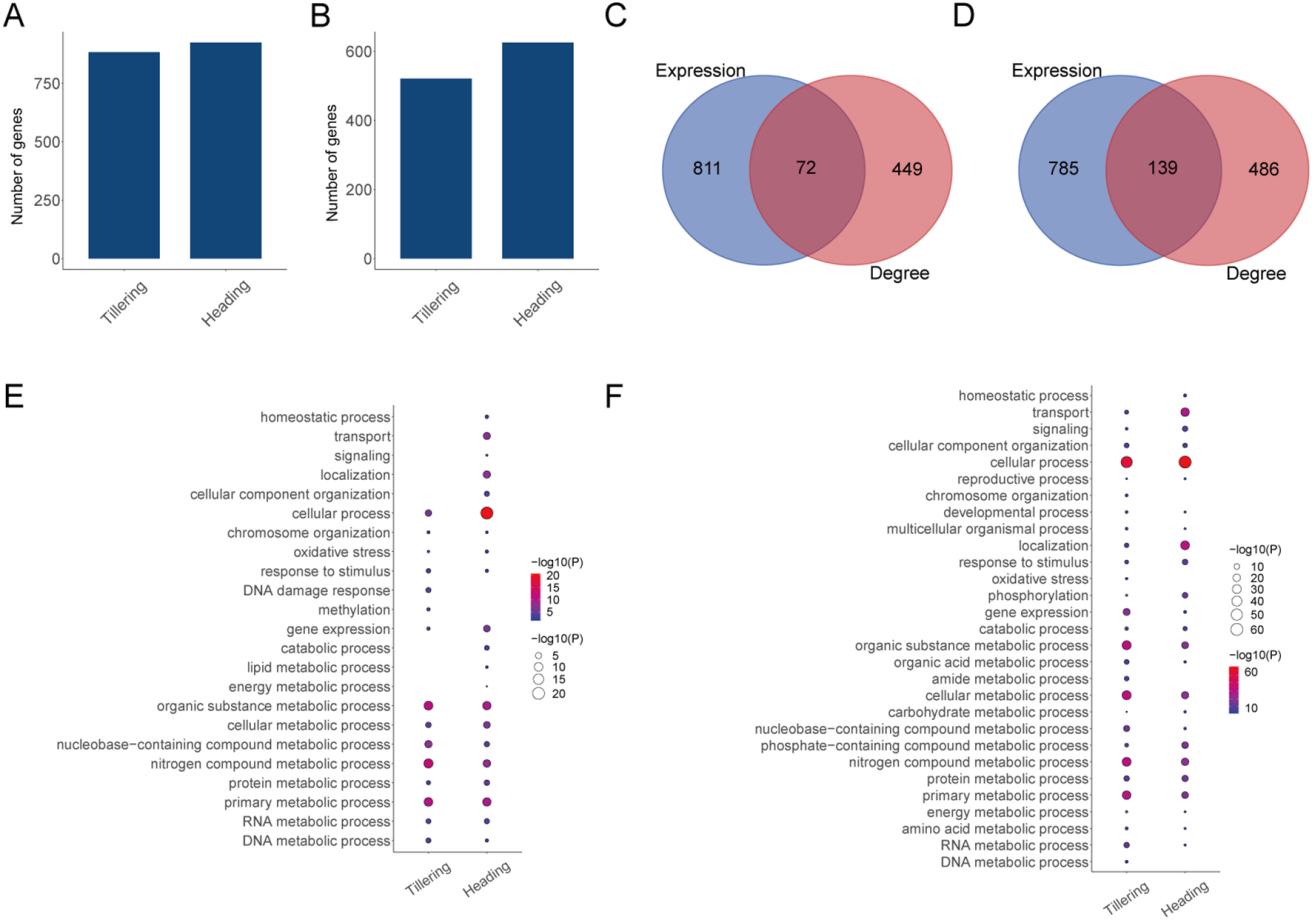
LET-related genes and their biological functions. **(A)** The number of LET-related genes (expression level). **(B)** The number of LET-related genes (degree). **(C)** The overlap of LET-related genes from expression levels and degrees in the tillering stage. **(D)** The overlap of LET-related genes from expression levels and degrees in the heading stage. **(E)** The biological processes of LET-related genes from expression levels. The size and color of the bubbles denote the negative logarithm of the P-values. **(F)** The biological processes of LET-related genes from degrees.

According to Pearson correlation analysis, the expression levels of 883 genes were found to be correlated with LET ( |PCC|>0.5 and p<0.05 ) during the tillering stage, while the expression levels of 924 genes were correlated with LET ( |PCC|>0.5 and p<0.05 ) during the heading stage (**Figure 2A**). The degrees of 521 genes were found to be correlated with LET ( p<0.05 ) during the tillering stage, while the degrees of 625 genes were correlated with LET ( p<0.05 ) during the heading stage (**Figure 2B**). Therefore, the above genes were identified as LET-related genes. Of note, the number of LET-related genes during the heading stage is greater than during the tillering stage. Moreover, during the tillering stage, the expression levels and degree of 72 genes are both correlated with LET (**Figure 2C**); during the heading stage, the expression levels and degree of 139 genes are both correlated with LET (**Figure 2D**).

GO enrichment analysis of these LET-related genes reveals their primary involvement in metabolic process, response to stimulus, oxidative stress, phosphorylation, homeostatic process, methylation, developmental process, and reproductive process, etc. (**Figures 2E, F**). Where, metabolic processes include DNA metabolic process, RNA metabolic process, energy metabolic process, amino acid metabolic process, protein metabolic process, lipid metabolic process, organic substance metabolic process, and nitrogen compound metabolic process, etc. (**Figures 2E, F**). Note that DNA damage response occurs during the tillering stage (**Figure 2E**). For LET-related genes identified by expression levels, more biological processes were enriched during the heading stage. While for LET-related genes identified by degrees, the biological processes were roughly the same across both developmental stages.

The KEGG enrichment analysis reveals that LET-related genes are involved in a series of metabolic pathways, including RNA degradation, starch and sucrose metabolism, protein processing in the endoplasmic reticulum, glyoxylate and dicarboxylate metabolism, and the proteasome (**Supplementary Table S4**).

### 3.2 The hubs in LET-related genes

**Figure 3:**
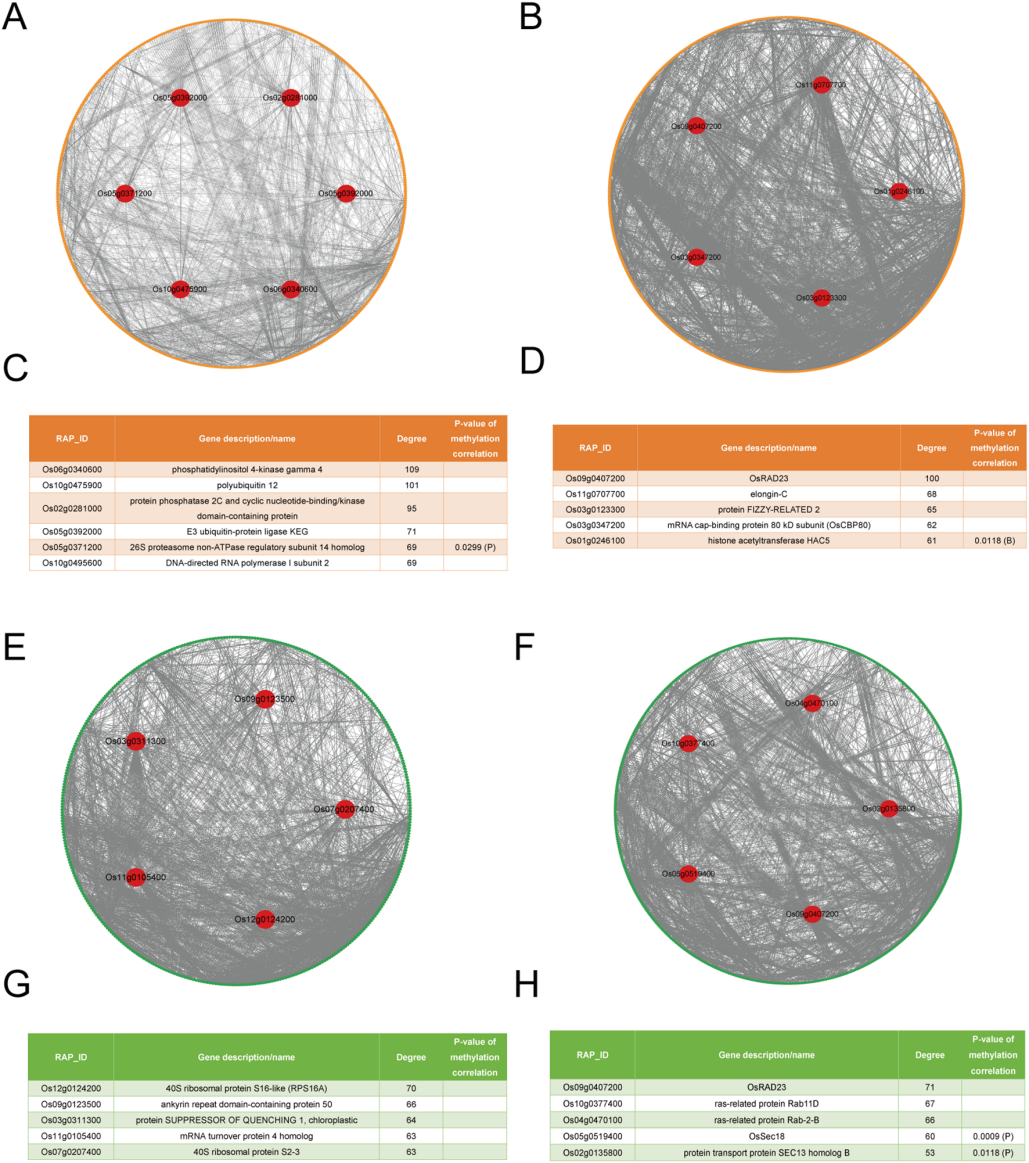
The PPI networks and hubs of LET-related genes. **(A)** The PPI networks of LET-related genes from expression levels in the tillering stage. Yellow circles represent LET-related genes identified through expression levels, green circles represent LET-related genes identified through degrees, and red circles represent hub genes. **(B)** The PPI networks of LET-related genes from expression levels in the heading stage. **(C)** The hubs in LET-related genes from expression levels in the tillering stage. The last column shows the P-values for the correlation between the expression levels (degrees) of radiation-responsive genes and their corresponding methylation levels (degrees). Blank values indicate non-significant correlations. “P” represents the promoter, and “B” represents the gene body. **(D)** The hubs in LET-related genes from expression levels in the heading stage. **(E)** The PPI networks of LET-related genes from degrees in the tillering stage. **(F)** The PPI networks of LET-related genes from degrees in the heading stage. **(G)** The hubs in LET-related genes from degrees in the tillering stage. **(H)** The hubs in LET-related genes from degrees in the heading stage.

Using STRING, four PPI networks were constructed, and four sets of hub genes were identified (**Figure 3**). During the tillering stage, there are two sets of hub genes identified. The first set, identified by expression levels, includes: Os06g0340600, Os10g0475900, Os02g0281000, Os05g0392000, Os05g0371200, and Os10g0495600 (**Figures 3A, C**). The second set, identified by degree, includes: Os12g0124200, Os09g0123500, Os03g0311300, Os11g0105400, and Os07g0207400 (**Figures 3E, G**). During the heading stage, there are also two sets of hub genes identified. The first set, identified by expression levels, includes: Os09g0407200, Os11g0707700, Os03g0123300, Os03g0347200, and Os01g0246100 (**Figures 3B, D**). The second set, identified by degree, includes: Os09g0407200, Os10g0377400, Os04g0470100, Os05g0519400, and Os02g0135800 (**Figures 3F, H**). Of note, Os11g0105400 and Os07g0207400 have the same degree; Os05g0371200 and Os10g0495600 also have the same degree. Of note, the FPKM values of all hub genes are shown in **Supplementary Table S5**.

Remarkably, the expression levels (degrees) of Os05g0371200 ( p=0.0299 ), Os01g0246100 ( p=0.0118 ), Os05g0519400 ( p=0.0009 ), and Os02g0135800 ( p=0.0118 ) are significantly correlated with their corresponding methylation levels (degrees) (**Figure 3**).

### 3.3 LET-related genes affected by methylation

**Figure 4:**
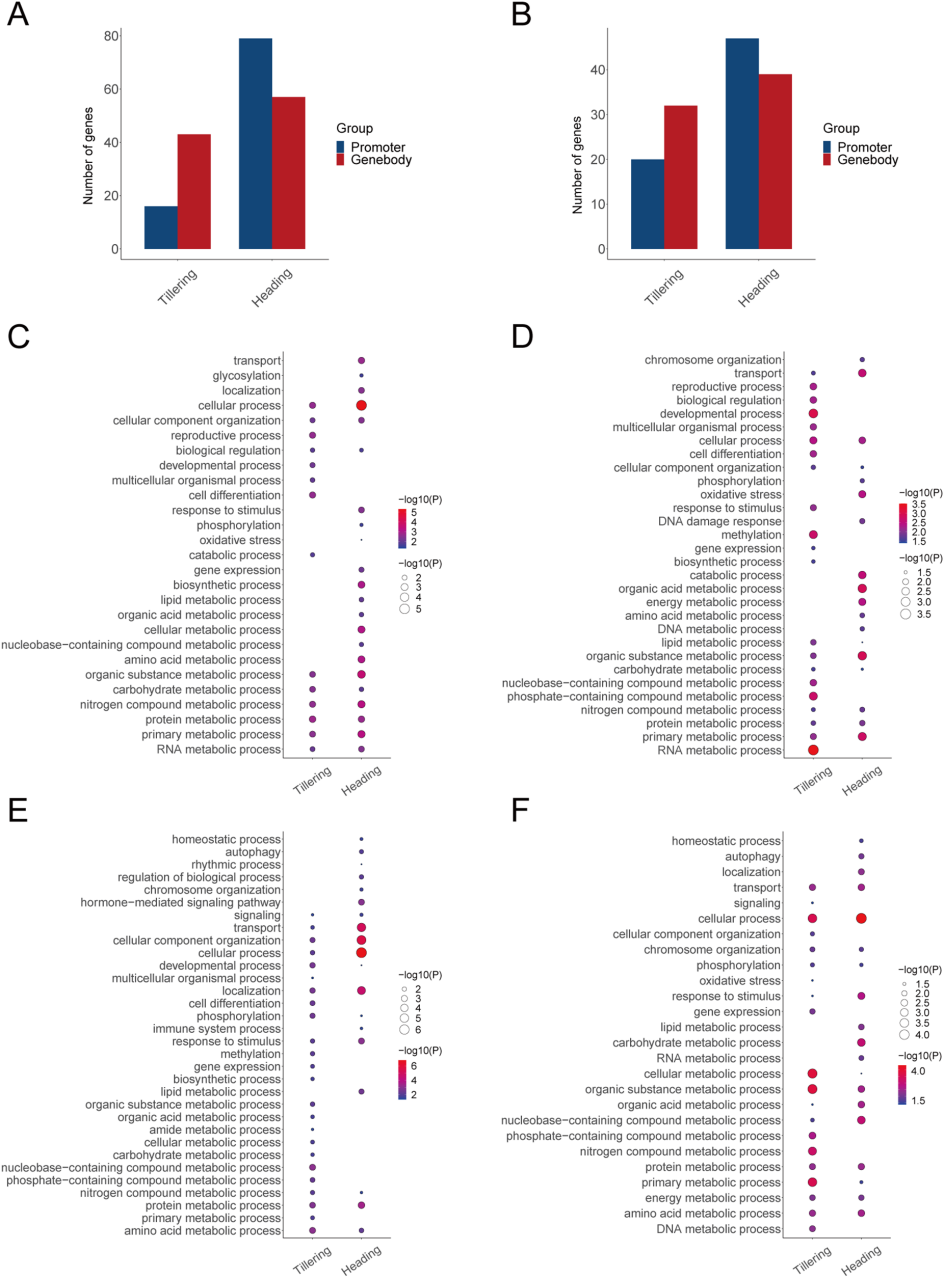
LET-related genes affected by methylation. **(A)** The number of LET-methylation genes (expression levels). **(B)** The number of LET-methylation genes (degrees). **(C)** The biological processes of LET-methylation genes from expression levels in promoters. **(D)** The biological processes of LET-methylation genes from expression levels in gene bodies. **(E)** The biological processes of LET-methylation genes from degrees in promoters. **(F)** The biological processes of LET-methylation genes from degrees in gene bodies.

To investigate the effects of methylation on the body’s LET response, we conducted a Pearson correlation analysis between the expression levels (gene degrees) of LET-related genes and their corresponding methylation levels (methylation degrees). During the tillering stage, the expression levels of 16 genes are correlated with the methylation levels in promoters, and the expression levels of 43 genes are correlated with the methylation levels in gene bodies. During the heading stage, the expression levels of 79 genes are correlated with the methylation levels in promoters, and the expression levels of 57 genes are correlated with the methylation levels in gene bodies (**Figures 4A, B**). Moreover, during the tillering stage, the degrees of 20 genes are correlated with the methylation degrees in promoters, and the degrees of 32 genes are correlated with the methylation degrees in gene bodies. During the heading stage, the degrees of 47 genes are correlated with the methylation degrees in promoters, and the degrees of 39 genes are correlated with the methylation degrees in gene bodies (**Figures 4A, B**). Therefore, the above genes were identified as LET-methylation genes. Notably, during the tillering stage, the number of LET-methylation genes in the gene bodies is higher than in the promoters, whereas, in the heading stage, the opposite is true. Overall, the number of LET-methylation genes is higher in the heading stage compared to the tillering stage.

According to the GO enrichment analysis, LET-methylation genes are mainly involved in DNA damage response, response to stimulus, oxidative stress, phosphorylation, developmental process, immune system process, and various metabolic processes (such as DNA metabolic process, RNA metabolic process, energy metabolic process, amino acid/protein metabolic process) (**Figures 4C–F**).

According to the ClueGO analysis, LET-methylation genes are mainly involved in various metabolic pathways, including carbohydrate metabolism, lipid metabolism, amino acid metabolism, protein metabolism, nucleotide metabolism, and vitamin and cofactor metabolism, etc. (**Table 2**). Additionally, they are also involved in oxidative phosphorylation, peroxisome, and plant-pathogen interaction pathway, etc. (**Table 2**).

**Table 2:** Pathways involving LET-methylation genes.

	Tillering stage	Heading stage
Promoter (expression)	Glycosphingolipid biosynthesis Spliceosome Proteasome	Oxidative phosphorylation Purine metabolism Pyrimidine metabolism Glycine, serine and threonine metabolism Cysteine and methionine metabolism Phenylalanine, tyrosine and tryptophan biosynthesis Glutathione metabolism Starch and sucrose metabolism N-Glycan biosynthesis Sphingolipid metabolism Aminoacyl-tRNA biosynthesis RNA transport Basal transcription factors Spliceosome Proteasome Protein processing in endoplasmic reticulum Plant-pathogen interaction
Gene body (expression)	Starch and sucrose metabolism Vitamin B6 metabolism RNA transport Spliceosome Proteasome Plant hormone signal transduction	Purine metabolism Valine, leucine and isoleucine degradation Proteasome Protein processing in endoplasmic reticulum Peroxisome
Promoter (degree)	Ascorbate and aldarate metabolism beta-Alanine metabolism Ribosome Basal transcription factors Plant hormone signal transduction Endocytosis	Glycine, serine and threonine metabolism Lipoic acid metabolism Ribosome RNA transport Proteasome Phosphatidylinositol signaling system Plant hormone signal transduction Endocytosis
Gene body (degree)	Glycolysis/Gluconeogenesis Valine, leucine and isoleucine degradation Lysine degradation Ribosome biogenesis in eukaryotes Basal transcription factors Plant hormone signal transduction Endocytosis	Pentose phosphate pathway Steroid biosynthesis Glycine, serine and threonine metabolism Carbon fixation in photosynthetic organisms Lipoic acid metabolism Porphyrin and chlorophyll metabolism Tropane, piperidine and pyridine alkaloid biosynthesis Ribosome Endocytosis

All KEGG pathways in the table were obtained through ClueGO analysis. “Expression” refers to LET-methylation genes identified based on the correlation of expression levels, while “degree” refers to LET-methylation genes identified based on the correlation of degrees.

### 3.4 LET-regression models and radiation-responsive genes

**Figure 5:**
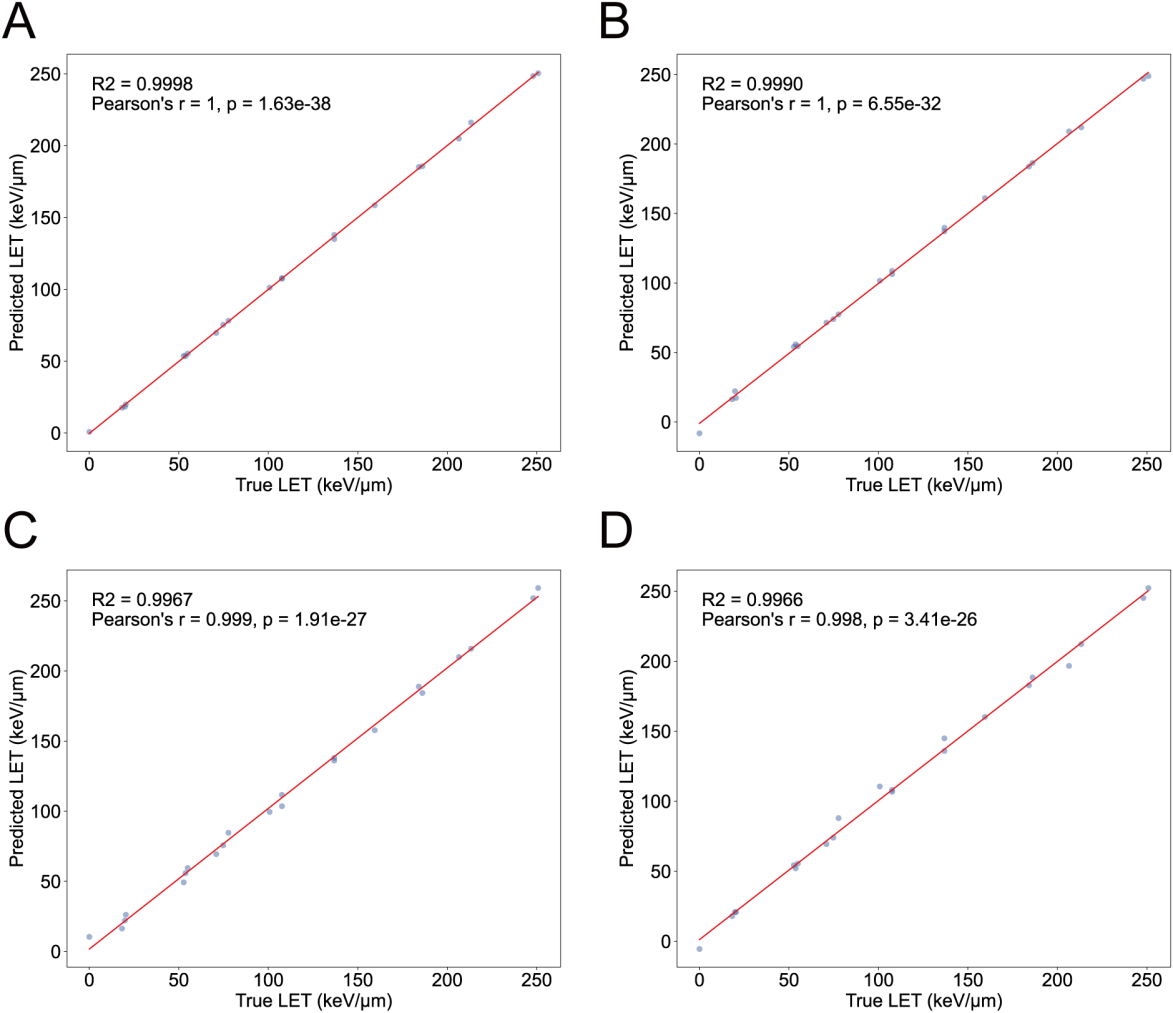
The performance of LET-regression models. **(A)** The performance of expression-model in the tillering stage. **(B)** The performance of expression-model in the heading stage. **(C)** The performance of degree-model in the tillering stage. **(D)** The performance of degree-model in the heading stage. The X-axis represents the true LET values, and the Y-axis represents the predicted LET values. Each point represents a sample.

During the tillering stage, the expression-model has an *R* ^2^ of 0.9998, with a PCC of 1 between the actual and predicted values ( p=1.63e−38 ); The degree-model has an *R* ^2^ of 0.9967, with a PCC of 0.999 between the actual and predicted values ( p=1.91e−27 ). During the heading stage, the expression-model has an *R* ^2^ of 0.9990, with a PCC of 1 between the actual and predicted values ( p=6.55e−32 ); The degree-model has an *R* ^2^ of 0.9966, with a PCC of 0.998 between the actual and predicted values ( p=3.41e−26 ) (**Figure 5**). Therefore, both the expression-models and the degree-models exhibit excellent regression performance.

During the tillering stage, the expression-model identified 20 radiation-responsive genes (**Table 3**), and the degree-model also identified 20 radiation-responsive genes (**Table 4**). During the heading stage, the expression-model identified 18 radiation-responsive genes (**Table 5**), and the degree-model identified 20 radiation-responsive genes (**Table 6**). Note that the FPKM values of all radiation-responsive genes are shown in **Supplementary Table S5**.

**Table 3:** Radiation-responsive genes of expression-model in tillering stage.

RAP_ID	Gene description/name	P-value of methylation correlation
Os01g0580100	Mannosyltransferase.	
Os01g0874700	patellin-3	
Os01g0881500	scarecrow-like protein 1	
Os01g0953500	uncharacterized LOC4325399	
Os02g0471500	Probable protein phosphatase 2C 14.	
Os03g0137500	Mitochondrial outer membrane protein porin 6 (VDAC6)	
Os03g0157900	Phosphoserine aminotransferase.	
Os03g0385900	Ribosomal protein S11 containing protein, expressed.	
Os03g0454300	–	
Os03g0662000	–	
Os03g0698800	Zinc finger CCCH domain-containing protein 24.	
Os04g0283900	–	
Os05g0243200	uncharacterized protein LOC4338198	0.0161 (Promoter); 0.0213 (Gene body)
Os07g0661100	N-acetylglucosaminephosphotransferase	
Os08g0200300	photosystem II 10 kDa polypeptide, chloroplastic	
Os09g0418000	CBL-interacting protein kinase 16 (CIPK16)	
Os09g0520100	uncharacterized LOC4347604	
Os12g0228500	glutathione S-transferase T3	
Os12g0538500	E3 ubiquitin-protein ligase MIEL1	
Os08g0335300	–	

The last column shows the P-values for the correlation between the expression levels of radiation-responsive genes and their corresponding methylation levels. Blank values indicate non-significant correlations.

**Table 4:** Radiation-responsive genes of expression-model in heading stage.

RAP_ID	Gene description/name	P-value of methylation correlation
Os01g0214400	uncharacterized LOC9269554	
Os01g0571000	mitochondrial carrier protein CoAc1	
Os02g0437800	vacuolar protein sorting-associated protein 45 homolog	0.0498 (Promoter)
Os02g0567100	Thioredoxin-like 4, chloroplastic	
Os04g0681900	Acyl-CoA-binding domain-containing protein 4 (ACBP4)	
Os04g0692750	SNF2 domain-containing protein ENL1-like	
Os05g0589700	inactive protein kinase SELMODRAFT_444075	
Os06g0158200	protein-tyrosine sulfotransferase	0.0434 (Promoter)
Os06g0316800	uncharacterized LOC4340876	
Os07g0529800	Protein translation factor SUI1 homolog (GOS2)	
Os07g0661600	E3 ubiquitin-protein ligase AIRP2	
Os08g0511900	uncharacterized LOC4346012	
Os09g0525600	plant UBX domain-containing protein 8	
Os09g0573100	uncharacterized LOC4347931	
Os10g0167500	nucleolar protein 12	
Os11g0526800	uncharacterized LOC9268504	
Os12g0156400	Dcp1-like decapping family protein	
Os04g0353200	hypothetical protein	

The last column shows the P-values for the correlation between the expression levels of radiation-responsive genes and their corresponding methylation levels. Blank values indicate non-significant correlations.

**Table 5:** Radiation-responsive genes of degree-model in tillering stage.

RAP_ID	Gene description/name	P-value of methylation correlation
Os09g0106700	Transcription factor MYB44	
Os09g0407200	Probable ubiquitin receptor RAD23 (OsRAD23)	
Os02g0533900	N-carbamoylputrescine amidase (CPA)	
Os06g0347100	acyl-coenzyme A oxidase 4, peroxisomal	
Os04g0289800	E3 ubiquitin-protein ligase ORTHRUS 2	
Os10g0155800	leucine-rich repeat receptor-like protein kinase PEPR2	
Os02g0181800	ubiquitin fusion degradation protein 1 homolog	
Os03g0412800	Glucose-6-phosphate 1-dehydrogenase	
Os11g0121400	Serine/threonine-protein kinase NAK	
Os10g0184300	V-type proton ATPase subunit a	
Os01g0166800	mini-chromosome maintenance complex-binding protein	
Os10g0118900	probable helicase MAGATAMA 3	
Os01g0590700	uncharacterized LOC9267860	
Os02g0175700	putative clathrin assembly protein At5g57200	0.0045 (Gene body)
Os11g0248200	la-related protein 6B	
Os03g0738000	uncharacterized LOC4334044	
Os09g0451800	uncharacterized LOC4347228	
Os12g0129000	uncharacterized LOC9272112	
Os10g0474800	long-chain-alcohol oxidase FAO1	
Os11g0640300	Leucine Rich Repeat family protein	

The last column shows the P-values for the correlation between the degrees of radiation-responsive genes and their corresponding methylation degrees. Blank values indicate non-significant correlations.

**Table 6:** Radiation-responsive genes of degree-model in heading stage.

RAP_ID	Gene description/name	P-value of methylation correlation
Os12g0299700	nucleolar protein 58	
Os12g0226900	NADP-dependent oxidoreductase P2	
Os05g0204900	serine/threonine-protein phosphatase 5	
Os08g0323000	uncharacterized LOC4345269	0.0043 (Promoter)
Os01g0858500	transcription initiation factor TFIID subunit 12	
Os08g0127700	Pre-mRNA-splicing factor SLU7	0.0083 (Gene body)
Os04g0376500	Eukaryotic translation initiation factor 3 subunit H.	
Os03g0776900	mitochondrial import inner membrane translocase subunit TIM14-3	
Os10g0580700	Ankyrin-2	
Os06g0486400	uncharacterized LOC4341068	
Os10g0524400	Phospholipase D beta 1	
Os11g0183700	SWI/SNF complex subunit SWI3C	
Os05g0182100	protein SLOW GREEN 1, chloroplastic	
Os04g0417600	uncharacterized LOC9268987	
Os04g0625600	BTB/POZ and MATH domain-containing protein 2	0.0175 (Promoter)
Os01g0886000	uncharacterized LOC4324886	
Os03g0157500	K+ channel tetramerisation domain containing protein	
Os02g0748100	RINT1-like protein MAG2	
Os06g0134800	putative transferase At4g12130, mitochondrial	
Os01g0531200	serine/threonine-protein phosphatase 7 long form homolog	

The last column shows the P-values for the correlation between the degrees of radiation-responsive genes and their corresponding methylation degrees. Blank values indicate non-significant correlations.

The expression levels (degrees) of several radiation-responsive genes are significantly correlated with their corresponding methylation levels (degrees): for instance, Os05g0243200 ( p=0.0161 in promoter; p=0.0213 in gene body) and Os02g0175700 ( p=0.0045 ) during the tillering stage; Os02g0437800 ( p=0.0498 ), Os06g0158200 ( p=0.0434 ), Os08g0323000 ( p=0.0043 ), Os04g0625600 ( p=0.0175 ), and Os08g0127700 ( p=0.0083 ) during the heading stage (**Tables 3**–**6**).

### 3.5 The biological functions of radiation-responsive genes

**Figure 6:**
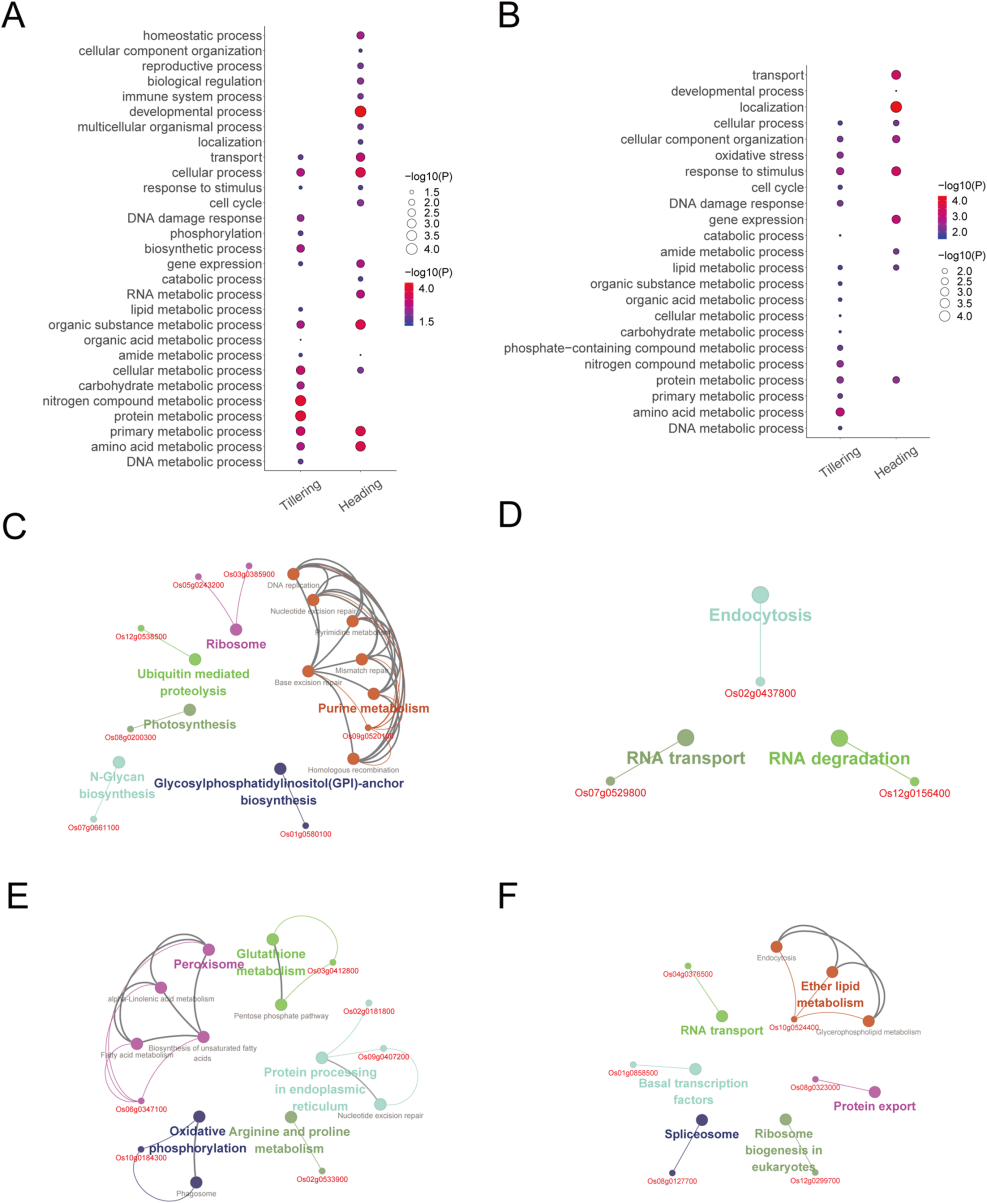
The biological functions of radiation-responsive genes. **(A)** The biological processes of radiation-responsive genes identified through expression-models. **(B)** The biological processes of radiation-responsive genes identified through degree-models. **(C)** The pathway of radiation-responsive genes in the tillering stage (expression-model). **(D)** The pathway of radiation-responsive genes in the heading stage (expression-model). **(E)** The pathway of radiation-responsive genes in the tillering stage (degree-model). **(F)** The pathway of radiation-responsive genes in the heading stage (degree-model).

To investigate the functions of the radiation-responsive genes mentioned above, we performed a GO enrichment analysis on these genes, and found that they were primarily involved in DNA damage response, cell cycle, immune system process, response to stimulus, and various metabolic processes (**Figures 6A, B**).

According to the ClueGO analysis, radiation-responsive genes are mainly involved in DNA repair (including base excision repair, mismatch repair, nucleotide excision repair, and homologous recombination), metabolic pathways (including nucleotide metabolism, lipid metabolism, carbohydrate metabolism, protein metabolism, and amino acid metabolism, etc.), oxidative phosphorylation, peroxisome, and photosynthesis, etc. (**Figures 6C–F**). Of note, DNA repair occurs during the tillering stage (**Figures 6C, E**).

## 4 Discussion

Although dose-dependent effects in the space environment have been reported (Zhang et al., 2024a; Zhang et al., 2024b), how the body responds to heavy ions with different LET values still requires further study. In this work, we used rice as a model organism to study the LET-dependent effects caused by heavy ions. The biostack was a commonly used method for radiobiological experiments in space (Bücker et al., 1984; Horneck, 1992; Reitz et al., 1995), which was used as a payload of the SJ-10 flying in a LEO of 252 km for 12.5 days. After the satellite returned to Earth, we obtained seeds with embryos hit by a single heavy ion through the biostack and detected the LET value of each heavy ion using CR-39. These space-returned seeds were cultivated through their full lifecycle in a controlled climate chamber and the leaves were collected at the tillering and heading stages for RNA-Seq and WGBS. Next, we designed a bioinformatics pipeline based on GA to comprehensively analyze the transcriptome and methylation data of rice under spaceflight conditions. LET-related genes and radiation-responsive genes were identified in this study, and further analysis of these genes could help explore the various effects of heavy ions on plants.

Remarkably, microgravity under spaceflight conditions can also have biological effects on rice. Since this study primarily focused on analyzing the LET effects of heavy ions, the main consideration was the impact of changes in LET on plants. Given that the flight duration was the same, there was minimal variation in microgravity among the different plants. Moreover, the germination and growth of our seeds took place on Earth, and the rice was not planted until 606 days after returning to Earth, so the effects of microgravity were likely minimal. Therefore, this paper did not provide a detailed analysis of the effects of microgravity. However, we must acknowledge that the LET effects observed in this study occurred under microgravity conditions, which was a characteristic of space radiation’s biological effects. The combined effects of LET and microgravity warrant further investigation.

### 4.1 Exploring the molecular changes caused by heavy ions from two perspectives

In this study, we explored the molecular changes caused by heavy ions from two perspectives. One perspective is the traditional approach, which involves observing changes in gene expression and methylation levels caused by heavy ions with different LET values and identifying genes associated with LET variations. Additionally, we also examined the changes from the perspective of gene networks. Previous studies suggested that employing molecular networks could reveal dysfunctional interactions or disordered regulation from a systemic standpoint (Huang et al., 2021). Therefore, we utilized LIONESS to construct three SSNs (gene-SSN, meth-promoter-SSN, meth-body-SSN) for each sample respectively. LIONESS is a method utilized for reverse engineering SSNs from aggregate networks (Kuijjer et al., 2019), and its application has been increasingly prevalent in recent times. By observing changes in gene degrees under different LET conditions, we could understand alterations in gene interaction patterns caused by heavy ions. Analyzing from two perspectives can yield complementary results, thereby providing a comprehensive explanation of biological changes under spaceflight conditions from systems biology. The experimental results indicate that both gene expressions and interaction patterns can reflect the molecular changes caused by heavy ions with different LET values. Based on gene overlap (**Figures 2C, D**), it is evident that two models (expression-model and degree-model) can identify unique genes from their respective perspectives. However, two models can also identify the same genes (**Figures 2C, D**), indicating that the expression levels and interaction patterns of certain genes will change under the impact of heavy ions. Currently, there is limited research on the changes in gene interaction patterns at the methylation level using the SSN method. To our knowledge, this is the first application of the SSNs to reveal the dysregulation of gene interaction patterns under spaceflight conditions in multi-omics studies.

### 4.2 Design of genetic algorithm

Essentially, identifying radiation-responsive genes from nearly twenty thousand genes is a process of feature selection. In our work, it involves identifying the gene combination that best predicts LET values, and evidently, these genes are radiation-responsive genes. The GA as a fundamental optimization tool has been widely used in feature selection tasks (Too and Abdullah, 2021). It can effectively enhance model performance while selecting the best feature combinations. In a recent study (Zhang et al., 2024a), we employed the GA to construct regression models for 301 spaceflight mouse samples, accurately predicting the absorbed dose for each sample. In that study, we also identified 20 radiation-responsive genes in the mice. However, due to the limited number of samples involved in the present study, directly applying previous methods may introduce a large number of false positive genes. Therefore, in this work, we optimized algorithm (Zhang et al., 2024a) by redesigning the initialization, crossover operator, and mutation operator. This enhancement enabled the algorithm to select a limited number of genes while maintaining regression performance, thereby improving the quality of identified radiation-responsive genes. From the experimental results, it is evident that both the expression-models and the degree-models exhibit excellent regression performance (*R* ^2^ close to 1) (**Figure 5**), demonstrating the reliability of our models.

### 4.3 Biological processes and pathways involving LET-related genes and radiation-responsive genes

Enrichment analysis indicates that LET-related genes and radiation-responsive genes are primarily involved in biological processes and pathways such as DNA damage and repair, oxidative stress, oxidative phosphorylation, and photosynthesis. DNA repair is an important biological process in plants exposed to space radiation. Dixit et al. exposed 10-day-old *Arabidopsis thaliana* seedlings to simulated GCR and evaluated the resulting transcriptomic changes (Dixit et al., 2023). Their findings revealed a dose-dependent transcriptomic response, with a notable upregulation of DNA repair pathways (Dixit et al., 2023). Transcriptomic analysis of *Arabidopsis thaliana* onboard the Space Shuttle also revealed expression changes in genes involved in DNA repair (Johnson et al., 2017). Of note, we found that DNA damage and repair primarily occurs during the tillering stage in this work. Spaceflight environment have been shown to generate reactive oxygen species (ROS) and induce oxidative stress in plants (Sugimoto et al., 2014). Zeng et al. also used SJ-10 to carry DN423 rice seeds, which were planted until the F2 generation after returning to Earth (Zeng et al., 2022b). They found that oxidative stress signals activated sugar signals to rebuild metabolic networks, enabling adaptation to spaceflight stress (Zeng et al., 2022b). Moreover, our previous study has shown that rice’s photosynthetic capacity decreases after spaceflight, with reductions in chlorophyll content during the trefoil, tillering, and maturity stages, and a decline in proteins related to key photosynthetic enzymes (Cui et al., 2019).

LET-related genes and radiation-responsive genes are also enriched in several metabolic processes and pathways, such as nucleic acid metabolic process (DNA metabolic process, RNA metabolic process, nucleotide metabolism), energy metabolic process, amino acid metabolic process, protein metabolic process, and lipid metabolic process, etc. A research indicated that spaceflight could lead to alterations in the tricarboxylic acid cycle (TCA) rate and amino acid metabolism pathway in rice (Zeng et al., 2021). Spaceflight also leads to the suppression of transcription, post-transcriptional modification, protein synthesis, protein modification, and degradation processes (Zeng et al., 2022a). Besides, both transcriptomic and proteomic analyses indicate altered lipid and ion intracellular transport during spaceflight (Olanrewaju et al., 2023). From the above analysis, it is evident that the LET-related genes and radiation-responsive genes identified in this study play important biological roles in rice responses to space stressors.

Recently, there have been some reports about other plants responding to different LET radiation. The study by De Francesco et al. showed that low-LET ionizing radiation (IR) (X-ray) stimulated the production of antioxidants, improving the plant defense and nutritional value of *Brassica rapa* (De Francesco et al., 2023). Volkova et al., through multi-omics analysis of barley, discovered that various types of ionizing radiation (gamma, electron, proton, neutron) (different LET) enhanced protein catabolism and resulted in a reduction in translation activity (Volkova et al., 2024). Moreover, the proton beam, with its unique DNA mutagenesis patterns based on its LETs, was expected to be a valuable tool for developing new mutant varieties of *Cymbidium* (Lee et al., 2015). Comparing with our results, it can be seen that IR induces similar biological effects in different plants, such as alterations in metabolic process, response to stimulus, and DNA damage. Additionally, LET is an important parameter influencing the biological effects induced by radiation.

### 4.4 Functional analysis of LET-related genes (hubs) and radiation-responsive genes

In the hubs of LET-related genes and radiation-responsive genes identified by this paper, many genes are performing essential functions in abiotic stress responses. For example, RAD23 (Os09g0407200)-related nucleotide excision pathway plays a critical role in the UV response (Feng et al., 2020). Os02g0281000 is a protein phosphatase type 2C (PP2C), which has regulatory functions in the abscisic acid (ABA)-signaling pathway (Chuong et al., 2021). Os03g0123300 is a Tiller Enhancer (TE), and a loss-of-function mutation in TE causes hypersensitivity to ABA and hyposensitivity to gibberellic acid in rice (Lin et al., 2015). OsMSRFP (Os12g0538500) is an active E3 ligase, and knockout of OsMSRFP leads to rice salt tolerance (Xiao et al., 2022). Os02g0567100 is a tetratricopeptide-repeat thioredoxin-like (TTL) protein. Research has indicated a correlation between the TTL3 and the emergence and subsequent development of lateral roots (LR) (Xin et al., 2022). LRs are crucial elements at the interface between plants and soil, playing a significant role in stress resilience (Xin et al., 2022). In *Arabidopsis thaliana*, six ACBPs participate in development and stress responses (the name of Os04g0681900 is OsACBP4) (Meng et al., 2014). Os06g0316800 is a glycine-rich RNA-binding protein (GRP) that has been proven to play crucial roles in plant responses to environmental stressors (Kim et al., 2024). Os07g0529800 (OseIF1) has a central function in salt-stress adaptation in rice by regulating ion accumulation and the intracellular redox status (Diédhiou et al., 2008). Aminoacylase-1 (Os08g0511900) is a zinc-binding enzyme whose activity is suppressed by salt, drought, jasmonic acid, and salicylic acid, while it is stimulated by abscisic acid and ethylene (Chen et al., 2021). This suggests its potential involvement in stress responses and plant growth (Chen et al., 2021). As a substrate protein of the rapidly stress-induced mitogen-activated protein kinase MPK3, *Arabidopsis thaliana* transcription factor MYB44 (Os09g0106700 in rice) likely acts at the front line of stress-induced re-programming (Persak and Pitzschke, 2014). Genome-wide association studies (GWAS) have shown that the splice variants of OsRAD23 exhibit significant divergence among the variants in terms of shoot growth under salt stress conditions (Yu et al., 2021). Os03g0412800 is a glucose-6-phosphate 1-dehydrogenase, which is associated with the response to salt stress (Luo et al., 2018). Os10g0184300 is a vacuolar-type adenosine triphosphatase (V-ATPase), which is implicated in multiple stress responses as well as physiological processes such as growth, development, and morphogenesis (Wang et al., 2021). Serine/threonine phosphatase 5 (AtPP5, Os05g0204900) plays a pivotal role in heat stress resistance (Park et al., 2011). The expression of eIF3h is upregulated by high temperatures (Chen et al., 2019). Transgenic studies have demonstrated that suppressing the expression of rice PLD beta I (Os10g0524400) leads to reduced sensitivity to exogenous ABA during seed germination (Li et al., 2007). Therefore, genes that respond to heavy ions also play roles in other abiotic stresses, suggesting that the pathways through which plants resist stress may have similarities. Furthermore, other radiation-responsive genes identified in this study have not been found to play roles in resisting other stressors, suggesting that these genes may be specific to responding to heavy ions.

### 4.5 The role of DNA methylation in rice’s resistance to stressors caused by heavy ions

Studies have demonstrated that the spaceflight environment can significantly induce heritable epigenetic changes in rice, particularly altering cytosine methylation patterns and activating transposable elements (Ou et al., 2010). However, the patterns of methylation changes caused by heavy ions with different LET values in rice, along with their effects on gene expression, have not been documented. Therefore, we examined the methylation changes of LET-related genes and radiation-responsive genes from the perspectives of methylation levels and interaction patterns, identifying genes potentially regulated by methylation. Note that this is the first study to investigate the methylation changes in rice under spaceflight conditions from the perspective of individual gene networks (SSNs). The analysis results indicate that LET-methylation genes are primarily involved in various metabolic pathways, oxidative phosphorylation, peroxisome functions, etc. (**Figures 4C–F**), which are similar to the functions associated with LET-related genes and radiation-responsive genes. This suggests that DNA methylation plays a regulatory role in multiple important biological processes and pathways during plants’ resistance to stressors caused by heavy ions.

We further investigated the functions of radiation-responsive genes (LET-related hub genes) potentially regulated by methylation (**Table 3**–**6**). For instance, overexpressing OsSec18 (Os05g0519400) in rice resulted in reduced plant height and 1000-grain weight, and it also modified the morphology of the protein bodies (Sun et al., 2015). Sec13p (Os02g0135800), as a part of the COPII complex, is involved in protein transport from the endoplasmic reticulum to the Golgi apparatus (Barlowe et al., 1994). This process may play an important role in plants’ responses to abiotic stress (Liu and Howell, 2010). Os01g0246100 (HAC5) is a histone acetyltransferase, and studies have shown that HACs are involved in the ethylene signaling pathway (Li et al., 2014). Os08g0127700 is a pre-mRNA-splicing factor that primarily participates in the RNA splicing process and is an essential component of RNA processing. Os04g0625600 is a BTB/POZ and MATH domain-containing protein, and this type of protein can enhance the growth and development of rice under salt stress (Ullah et al., 2023). OsANTH3 (Os02g0175700)-mediated endocytosis is important for rice pollen germination (Lee et al., 2021). Therefore, these radiation-responsive genes (LET-related hub genes) regulated by methylation not only participate in the response to abiotic stress but also play important roles in the regulation of plant phenotypes (such as plant height, grain weight, and pollen germination), epigenetic regulation, RNA processing, etc.

## 5 Conclusion

By developing a bioinformatics pipeline based on SSNs and GA, this study comprehensively analyzed the biological effects caused by heavy ions in rice. Both gene expressions and interaction patterns can reflect the molecular changes caused by heavy ions with different LET values. LET-related genes and radiation-responsive genes were identified, primarily involved in DNA damage and repair, oxidative stress, photosynthesis, nucleic acid metabolism, energy metabolism, amino acid/protein metabolism, and lipid metabolism, etc. DNA methylation plays a crucial role in responding to heavy ions stressors and regulates the aforementioned processes.

## Data Availability

The datasets presented in this study can be found in online repositories. The names of the repository/repositories and accession number(s) can be found below: https://ngdc.cncb.ac.cn/gsa, CRA013651.
