# Supplementary material for: Using single-sample networks and genetic algorithms to identify radiation-responsive genes in rice affected by heavy ions of the galactic cosmic radiation with different LET values
Source: Front Plant Sci. 2024 Nov 8;15:1457587. doi: 10.3389/fpls.2024.1457587 (PMC11581881; doi:10.3389/fpls.2024.1457587)
Supplement: Supplementary file 1 [file Table1.docx]

**Supplementary Table 1. LET values of 103 rice seeds.**

| **Plant ID** | **LET (keV/μm)** |
| --- | --- |
| R30-B6-6-3-S | 10.0083 |
| R30-C2-1-1-S | 10.0571 |
| R30-C4-3-2-T | 11.1603 |
| R30-B6-9-1-T | 12.7932 |
| R30-B3-4-4-S | 13.3844 |
| R30-B1-3-3-T | 13.7613 |
| R30-B3-2-2-S | 14.0148 |
| R30-C8-1-5-T | 14.3286 |
| R30-C8-3-2-T | 14.9241 |
| R30-C8-9-3-T | 14.9241 |
| R30-C4-3-5-S | 17.7077 |
| R30-C8-2-5-S | 18.2679 |
| R30-C3-3-1-S | 18.449 |
| R30-C4-9-5-T | 18.5981 |
| R30-A5-8-2-T | 19.8511 |
| R30-C2-7-1-T | 20.0042 |
| R30-B6-2-2-S | 20.4151 |
| R30-B3-3-4-T | 21.4588 |
| R30-B1-11-1-T | 21.6267 |
| R30-B6-7-4-T | 22.2488 |
| R30-C3-4-2-S | 23.4311 |
| R30-C2-9-1-T | 24.1381 |
| R30-B6-5-4-S | 25.433 |
| R30-C8-6-3-T | 27.8877 |
| R30-A5-9-3-T | 29.3177 |
| R60-C3-3-2-S | 33.3875 |
| R60-C8-5-5-T | 35.4593 |
| R60-A8-1-2-T | 36.0492 |
| R60-C4-5-5-S | 37.0248 |
| R60-B3-2-3-T | 37.0574 |
| R60-A5-7-2-S | 37.2939 |
| R60-C3-3-3-T | 38.2018 |
| R60-B6-2-6-S | 38.9328 |
| R60-A8-3-2-T | 40.034 |
| R60-A12-3-2-T | 40.8941 |
| R60-A4-6-5-T | 43.053 |
| R60-A4-7-5-S | 43.053 |
| R60-A8-9-3-T | 44.7289 |
| R60-A4-5-4-S | 46.8989 |
| R60-A12-6-3-S | 47.1964 |
| R60-C4-9-1-T | 48.5435 |
| R60-A8-2-2-T | 51.4118 |
| R60-A4-7-1-S | 52.6354 |
| R60-C8-7-3-S | 52.7435 |
| R60-C8-9-2-T | 52.7435 |
| R60-B3-2-4-S | 53.5476 |
| R60-A4-8-4-T | 53.7153 |
| R60-A4-4-6-T | 54.9614 |
| R100-B6-3-2-T | 67.9567 |
| R100-C8-4-4-S | 67.9767 |
| R100-C4-8-5-T | 68.319 |
| R100-A5-6-1-S | 69.9156 |
| R100-A8-2-5-S | 70.471 |
| R100-B6-6-5-T | 70.9111 |
| R100-A8-8-4-T | 71.6914 |
| R100-A5-2-2-S | 74.2621 |
| R100-A8-6-1-T | 74.8003 |
| R100-B6-9-3-T | 76.0359 |
| R100-A12-5-2-S | 77.0058 |
| R100-A5-3-3-T | 77.7099 |
| R100-B1-6-3-T | 80.3028 |
| R100-B1-7-3-S | 80.5287 |
| R100-C3-5-5-S | 81.3645 |
| R100-A12-6-2-S | 82.0746 |
| R100-C3-6-3-T | 82.6599 |
| R100-B6-5-5-T | 90.8448 |
| R100-C3-7-1-S | 92.0858 |
| R100-C2-8-2-S | 95.8086 |
| R150-A4-8-2-S | 107.6213 |
| R150-A8-4-4-T | 107.6414 |
| R150-A8-5-3-T | 107.6414 |
| R150-A12-3-1-S | 115.9728 |
| R150-A12-8-3-T | 127.381 |
| R150-C3-6-2-S | 128.7918 |
| R150-C3-1-1-T | 136.8396 |
| R150-B3-9-3-S | 136.8545 |
| R150-A8-9-2-T | 139.5567 |
| R150-C3-9-5-S | 142.5739 |
| R150-A5-4-1-T | 148.6806 |
| R200-B1-1-2-T | 157.3876 |
| R200-A5-9-1-S | 159.4854 |
| R200-B6-3-6-T | 162.0942 |
| R200-C3-5-2-S | 170.9337 |
| R200-A8-7-2-S | 182.7917 |
| R200-A5-10-1-T | 184.0827 |
| R200-C3-4-1-T | 186.1181 |
| R200-B6-4-4-S | 188.4816 |
| R200-B6-2-3-S | 192.0439 |
| R200-B1-4-1-T | 194.201 |
| R300-B3-4-2-S | 206.4886 |
| R300-B3-1-1-T | 213.3132 |
| R300-A8-3-4-S | 238.1009 |
| R300-B6-9-4-T | 248.0167 |
| R300-B2-6-1-S | 250.8219 |
| R300-B6-3-1-S | 285.7351 |
| R300-A8-3-3-T | 311.5831 |
| R300-A8-5-2-T | 374.5766 |
| R300-C3-5-3-S | 377.6362 |
| R300-C3-9-2-T | 458.9479 |
| R300-C4-2-4-S | 673.7153 |
| R300-C8-5-3-T | 838.7451 |
| R300-B6-8-6-S | 851.5406 |
| R300-C4-7-2-T | 1092.1017 |
