# Supplementary material for: Using single-sample networks and genetic algorithms to identify radiation-responsive genes in rice affected by heavy ions of the galactic cosmic radiation with different LET values
Source: Front Plant Sci. 2024 Nov 8;15:1457587. doi: 10.3389/fpls.2024.1457587 (PMC11581881; doi:10.3389/fpls.2024.1457587)
Supplement: Supplementary file 4 [file Table4.docx]

**Supplementary Table 4. The KEGG pathways of LET-related genes.**

| **ID** | **Description** | **P-value** | **Method** | **Stage** |
| --- | --- | --- | --- | --- |
| osa03018 | RNA degradation | 0.037444498 | Expression | Tillering |
| osa00500 | Starch and sucrose metabolism | 0.005831408 | Expression | Heading |
| osa04141 | Protein processing in endoplasmic reticulum | 0.042641542 | Expression | Heading |
| osa00630 | Glyoxylate and dicarboxylate metabolism | 0.034943246 | Degree | Tillering |
| osa03050 | Proteasome | 1.38E-05 | Degree | Heading |
| osa04141 | Protein processing in endoplasmic reticulum | 0.013036408 | Degree | Heading |
| osa04075 | Plant hormone signal transduction | 0.041289173 | Degree | Heading |
